# *Tetranychus evansi* (Tetranychidae) spider mites now a major solanaceous crop pest in Côte d’Ivoire

**DOI:** 10.1007/s10493-026-01125-y

**Published:** 2026-03-14

**Authors:** Emilie Deletre, Chloé Latapie, Alain Migeon, Philippe Auger, Nadia Larpin, Samuel Laboisse, Thibaud Martin

**Affiliations:** 1 HortSys, CIRAD, Université de Montpellier, Montpellier, France; 2 CSRS, Abidjan, Côte d’Ivoire; 3 ISTOM, Angers, France; 4 CBGP, INRAe, Montpellier, France; 5 Campus de Baillarguet, Montferrier sur Lez, 34980 Montpellier, France

**Keywords:** Spider mite, Solanacae, Chemical control, Phytoseiid mites

## Abstract

The West African market gardening sector has been plagued in recent decades by phytophagous mite damage to solanaceous crops. Recent studies in Benin and Burkina Faso have confirmed West African outbreaks of red spider mites (*Tetranychus evansi*), a novel mite species native to South America, which has now virtually replaced local species. This study aimed to identify the different mite species infesting solanaceous crops in Côte d’Ivoire, while assessing their abundance and mapping their distributions. Tomato, eggplant and African eggplant crops were sampled along a north-south transect. Structured interviews were conducted to gather information on the crop protection practices. We confirmed the presence of the invasive species, *Tetranychus evansi*, with higher densities noted in the north, where the hot dry climatic conditions were suitable for its development. Local *Tetranychus urticae* and *Polyphagotarsonemus latus* species were not very abundant. The highest spider mite diversity was observed in the Abidjan area, where two new species of the *T. urticae* group were found. Very few Phytoseiidae predatory mites were present, even in plots that had barely been treated or not at all. Five species were identified: *Neoseiulus barkeri*, *Neoseiulus teke*,* Amblyseius swirskii*,* Amblyseius tamatavensis*, and *Paraphytoseius horrifer.* These predatory phytoseiid species seemed unable to control *T. evansi* populations. According to farmers, abamectin was the only pesticide effective for controlling these pests. To reduce the chemical control intensity, biological control based on the introduction and dissemination of a specific predatory mite such as *Phytoseiulus longipes* could be considered on a national and regional scale.

**Supplementary Information:**

The online version contains supplementary material available at 10.1007/s10493-026-01125-y.

## Highlights

*Tetranychus evansi* has now virtually replaced local species in Cote d’Ivoire.

*Tetranychus evansi* is found with higher densities noted in area with hot dry climatic conditions.

Five species of phytoseiulus predatory mite were identified.

Abamectin was the only effective pesticide according to farmers.

**Supplementary Information:**

The online version contains supplementary material available at 10.1007/s10493-026-01125-y.

## Introduction

In West Africa, vegetable crops—particularly those of the Solanaceae family—are prone to severe attacks by phytophagous mites. Yet scant studies have focused on these pests in the region despite their high abundance (Adetonah et al. 2011). These mites mainly belong to the Tetranychidae family, including the most well-known species, *Tetranychus urticae*, which feeds on nearly 1,200 plant species (Migeon and Dorkeld 2022), and can infest all vegetable crops (okra, cucumbers, beans, lettuce, etc.). This cosmopolitan two-spotted spider mite species has been identified in 124 countries (Migeon and Dorkeld 2022). It can breed and develop under a range of temperature conditions, while achieving optimal development between 23 and 30 °C and at < 50% relative humidity (INRAe, 2018). Generational turnover is therefore very rapid, especially during hot weather. Over the past 20 years, *Tetranychus evansi*, native to South America, has been found in various regions of Africa, from Zimbabwe in 1979 to Benin in 2006, where it has caused major damage to solanaceous crops (*Solanum lycopersicum*,* Solanum macrocarpon* and *Capsicum annuum*) and *Amaranthus cruentus* (Gutierrez 1989; Saunyama and Knapp 2003; Adango et al. 2006; Azandémè Hounmalon et al. 2018a). This pest species has a relatively fast-paced lifecycle under optimal temperature conditions (~ 31 °C), thus explaining why it incurs such heavy economic loss in vegetable cropping systems (Bonato 1999). The two weaver spider mite species *T. evansi* and *T. urticae* can have a similar damaging impact on plants. The infested plant symptoms include the development of small white spots on the leaf surface caused by feeding punctures, which may lead to leaf drop in the event of outbreaks of these mites. The webs they spin on leaves and stems are typical of these two species and serve to protect them from some predators and sometimes even pesticides (INRAe, 2018). Other spider mite species have also been previously noted on solanaceous crops in West Africa, including *Tetranychus neocaledonicus*, which has been found on a range host plants such as *Capsicum annuum*,* Solanum aethiopicum* and *Solanum lycopersicum* (Migeon and Dorkeld 2022), and in Côte d’Ivoire as early as 1986 (Migeon 2021). *Tetranychus ludeni* is another cosmopolitan species that now flourishes throughout Africa and Asia. It has been reported in Benin on *Solanum aethiopicum*, *Solanum macrocarpon* and *Amaranthus cruentus* (Adango et al. 2006). Mites of the Tarsonemidae family, especially *Polyphagotarsemus latus*, are also prevalent in tropical areas, particularly in coastal regions under high relative humidity conditions (Gutierrez 1989). These mites primarily cause leaf deformation and brownish patches on the fruit tissues (Blancard and Ryckewaert 2021).

Predatory mites of the Phytoseiidae family are the main natural enemies of pest mites. Several species have been identified in West Africa, including the generalist species *Amblyseius swirskii* and *Neoseiulus barkeri*, which have been identified in Ghana and Benin (Azandémè-Hounmalon et al. 2018b). Laboratory findings have revealed that *A. swirskii* can breed on *T. urticae*,* T. evansi* and *P. latus* mites (Momen and Elsaway 1993; van Maanen et al. 2010; Onzo et al. 2012). *N. barkeri* has been identified as a potential predatory mite of *P. latus* in augmentative release trials on chili pepper (*Capsicum annuum*) crops (Fan and Petitt 1994). *Amblyseius tamatavensis is* another generalist predator mite that has been reported in Benin and Ghana on *Mallotus oppositifolius*,* Byrsocarpus coccineus*, banana (*Musa paradisiaca*) and oil palm (*Elaeis guineensis*) (Demite et al. 2022). Moreover, *Paraphytoeius horrifer* has been detected in Benin on *Mucuna* sp., as well as in Ghana (Demite et al. 2022). *Neoseiulus longispinosus* is known to feed on *T. urticae* prey (Song et al. 2016) and was recently detected for the first time in subSaharan Africa in a tomato cropfield in southern Benin (Azandémè-Hounmalon et al. 2022). *Phytoseiulus longipes* is another predator phytoseiid mite that feeds mainly on *T. evansi* prey (Ferrero 2006) and was introduced in Kenya in the 2010 s for natural regulation of *T. evansi* outbreaks (Kungu et al. 2018, 2020).

Apart from a few occasional investigations on phytophagous and predatory mite distributions in vegetable cropfields in Côte d’Ivoire in 1986 (Migeon 2021) and more recently in Burkina Faso (Drabo et al. 2023) and Benin (Adango et al. 2006, 2007, 2020; Onzo and Tossounon Yarou 2015; Azandémè-Hounmalon et al. 2018b), the diversity and distribution of pest and predatory mites have yet to be comprehensively studied in West Africa.

The present scoping study had two main objectives: (i) to characterise the diversity of phytophagous mites on Solanaceae, i.e. the main vegetable crops in Côte d’Ivoire, and associated predatory mites populations; and (ii) to assess the impacts of various agroecological conditions and crop protection practices of vegetable farmers on the abundance and diversity of the target mite species.

## Materials and methods

### Study areas and plant material

The study was carried out in four large urban and semiurban areas of Côte d’Ivoire: Abidjan (number of sampling sites (n) = 7), Yamoussoukro (*n* = 10), Bouaké (*n* = 25) and Korhogo (*n* = 11) during the dry season in April-May 2022, i.e. a period that is highly conducive to mite development. From the list of producers involved in the Marigo Project, 100 producers were randomly contacted to identify those cultivating solanaceous crops. Among them, 53 producers were growing solanaceous crops and were available for the survey and sampling. Accordingly, a total of 53 field plots were sampled, focusing mainly on three solanaceous crops: *Solanum lycopersicum* (tomato, *n* = 20), *Solanum melongena* (eggplant, *n* = 7) and *Solanum aethiopicum* (African eggplant, *n* = 26). The GPS coordinates of each sampling site were recorded (graphical abstract).

### Surveys

Interviews were conducted with farmers managing these various monitored plots based on an interview guide. These structured interviews (Appendix 1) collected data on the farmers’ agricultural practices so as to identify those that could potentially affect the diversity and/or abundance of predatory and pest mites. The following data were recorded:

- sampling area (Abidjan, Yamoussoukro, Bouaké and Korhogo).
- crop species (tomato, eggplant, African eggplant), and its growth stage (1–4 scale).
- last pesticide treatment date, pesticide usage frequency, family of pesticides applied (neonicotinoid, avermectin, pyrethroid, indoxacarb, organophosphate, biopesticide, other) and their brand name.
- fungicide usage.
- last watering date.
- extent of weed cover and height and last weeding date.
- horticultural crop diversity around the plot (lettuce, oil palm, okra, cashew, banana, pepper, tomato, eggplant, maize, amaranth, cabbage, zucchini, etc.),
- presence and density of spider mites and phytoseiid mites and extent of infestation symptoms.

Data on farmers’ perception of the phytophagous mite problem were also colected: recognition of the term ‘mite’, of mite infestation symptoms and of the link between mites and symptoms, knowledge on acaricides, and mite sightings in the current year or previous years. Photos of damage symptoms and mite pests were presented during the interviews (Appendix 2).

### Field plot sampling

The plots were sampled according to several criteria: (i) availability of the farmer; (ii) detection of mites on tomato and eggplant plants. The plots were initially checked using a magnifying glass by proceeding in a zigzag pattern through the plot so as to cover as much of the area as possible; (iii) variability in the situations encountered (survey interview data). These criteria were taken into account during the field surveys in order to take stock of the diversity of Tetranychidae and Phytoseiidae mites present in Côte d’Ivoire. 19 mite-infested plots were sampled out of the 53 monitored plots.

### Sample collection

Ten plants present in two 4–12 m dia. circles, depending on the crop plot size, were randomly sampled in each plot (Okoth et al. 2009). The largest circle had to cover the entire plot. Plants on the plot edges were not sampled so as to avoid edge effects. Five leaflets per tomato plant and three leaves per eggplant were sampled at different plant heights. The number of sampled leaves or leaflets was decided based on the leaf area of the harvested crop species. The samples were randomly divided into five batches per plot. Each batch was then placed in a plastic Ziplock bag containing a square of absorbent paper to absorb any moisture and then stored in a cooler. The bags were subsequently taken to the Swiss Centre for Scientific Research in Côte d’Ivoire (CSRS) laboratory in Adiopodoumé and stored in a refrigerator at 8–10 °C prior to analysis.

### Mite storage and counting

Mite counts were carried out using the washing-rinsing technique described by Fauvel and Cotton (1983). Each batch of samples was placed in a water-filled container with a drop of liquid dishwashing detergent. After 1 h, the mixture was filtered through a fine mesh sieve (100 μm). Under a binocular microscope, Tetranychidae, Tarsonemidae and Phytoseiidae mites were counted and distributed in different tubes filled with 90% ethanol, following the dichotomous keys of Pritchard and Baker (1955), Krantz and Walter (2009) and Flechtmann and Knihinicki 2002. For plots which had an excessive spider mite density (> 200 mites), 1/8 of the mite sample was counted after homogenisation on a circular filter.

### Specimen identification

For each sampled plot, all phytoseiid mites (males and females) and 25 spider mites per batch (20 females and 5 males) were identified under a phase contrast microscope in the acarology laboratory of the CBGP joint research unit in Montpellier (France). The tube contents were poured onto a 5 cm dia. fine mesh sieve (100 μm) to separate the specimens. The spider mites were immersed in 50% lactic acid for 24 h to clarify them for microscope observation. Under a binocular microscope, the mites were collected with a fine-tipped brush and placed on a slide in a drop of Hoyer’s mounting medium, then positioned with a needle according to their family or gender for identification. Female Phytoseiidae and Tetranychidae mites were placed head up and abdomen down, while male Tetranychidae mites were placed in a lateral position so as to be able to observe their distinctive genitalia (aedeagus) (Zannou et al. 2006, 2007; Moraes et al. 2007; Sahraoui 2012). Once the coverslip was fitted, the slides were maintained for a week in an oven at 50 °C to dry the mounting medium. Finally, the coverslips were sealed with a solvent-based varnish.

### Data analysis

All statistical analyses were performed using the R Studio (version 4.3.3) software package. A preliminary data analysis was carried out using factor analysis of mixed data (FAMD) (FactoMineR package) to identify variables (see surveys) that could impact the presence of spider mites, tarsonemid mites and phytoseiid mites and to check for correlations between variables, thereby facilitating the selection of variables for the statistical model. The presence of spider mites, tarsonemid mites and phytoseiid mites according to the FAMD-identified variables was analysed using a generalized linear model (GLM) with a binomial distribution (lme4, car and MASS packages).

FAMD followed by hierarchical clustering on principle components (HCPC) were performed to classify plots and identify variables correlated with the spider mite abundance rates. The latter abundance rates according to HCPC classifications were analysed using a GLM with a negative binomial distribution. A zero-inflated negative binomial regression model was used to explain phytoseiid and tarsonemid mite abundance rates according to the survey variables.

Finally, between-plot spider mite and phytoseiid mite abundance rates were compared using a GLM with a negative binomial distribution. Pairwise between-group comparisons were performed using Tukey’s *post hoc* test.

.

## Results

### Diversity of phytophagous and predator mite species

Seventeen of the nineteen plots from which leaves were sampled for laboratory assessment were found to be infested by *T. evansi*, four by *T. urticae*, six by other spider mites of the *T. urticae* group, along with two unidentified species sampled on two different plots (Table 1, appendix 3). 84% of the plots were mainly infested by *T. evansi.* A species of the *T. urticae* group was found to predominate in only one plot. These different spider mite species were all present in the Korhogo area. *T. urticae* was not detected in the three other sampling areas. The two unidentified species sampled in the Abidjan area were mostly present in the plots where they had been detected. The per-sample spider mite abundance varied markedly, with the distribution of these pests often being very heterogeneous in the plots where they were detected. In many cases, only a few areas within the plot were infested.

**Table 1:** Mite species and mean density (± SE) per sample from the different sampling plots in Côte d’Ivoire

Plot	Cluster	Mean tetranyque mite density/sample (± SE)			Tetranyque species	Mean phytoseiid mite density/sample (± SE)			Phytoseiid species	Mean tarsonemid mite density/sample (± SE)		
C3	1	8.0	±	8.0 abc^2^	*Tetranychus evansi*, Spider mite of the *T. urticae* groupc	18.0	±	8.0 abc	*Amblyseius tamatavensis*, *Neoseiulus teke*, *Paraphytoseius horrifer*, *Neoseiulus barkeri*	4.0	±	2.0 a
F6	1	34.0	±	14.6 ab	*Tetranychus evansi*	0.3	±	0.3 ab	*Neoseiulus barkeri*	0.0	±	0.0 a
F5	1	47.0	±	22.3 ab	*Tetranychus evansi*, Spider mite of the *T. urticae* group	0.6	±	0.4 a	*Neoseiulus barkeri*	0.0	±	0.0 a
B2	1	48.0	±	15.5 abc	*Tetranychus evansi*	0.0	±	0.0 abc		0.0	±	0.0 a
A1	2	15.3	±	6.2 a	non-identified specie 1	21.0	±	7.3 c	*Neoseiulus teke*, *Amblyseius tamatavensis*, *Paraphytoseius horrifer*, *Amblyseius swirskii*	0.0	±	0.0 a
E2	2	35.4	±	29.0 a	*Tetranychus evansi*	0.2	±	0.2 a	*Amblyseius swirskii*	0.0	±	0.0 a
U7	2	78.6	±	22.2 abcd	*Tetranychus evansi*	0.0	±	0.0 abc		0.0	±	0.0 a
O4	2	145.0	±	145.0 bcdefg	non-identified specie 2	16.0	±	6.0 abc	*Paraphytoseius horrifer*, *Neoseiulus teke*	4.0	±	1.8 a
D1	2	513.4	±	137.8 bcdef	*Tetranychus evansi*	0.0	±	0.0 abc		0.0	±	0.0 a
K7	2	993.2	±	917.4 defg	*Tetranychus evansi*	0.6	±	0.4 a	*Neoseiulus barkeri*	15.8	±	10.4 a
I2	3	716.0	±	392.9 cdefg	*Tetranychus evansi*, Spider mite of the *T. urticae* group	19.2	±	7.4 c	*Neoseiulus teke*, *Paraphytoseius horrifer*, *Neoseiulus barkeri*	0.0	±	0.0 a
R3	3	5854.3	±	1628.4 fg	*Tetranychus evansi*, Spider mite of the *T. urticae* group	0.0	±	0.0 abc		20.0	±	15.5 a
R4	3	9001.3	±	2053.5 fg	*Tetranychus evansi*, Spider mite of the *T. urticae* group	0.0	±	0.0 abc		17.7	±	13.7 a
T9	4	25.4	±	7.0 a	*Tetranychus evansi*, *Tetranychus urticae*	6.0	±	2.3 abc	*Amblyseius swirskii*, *Neoseiulus teke*, *Neoseiulus barkeri*	21.8	±	9.1 a
P1	4	76.0	±	58.9 abcde	*Tetranychus evansi*	0.0	±	0.0 abc		0.0	±	0.0 a
T6	4	124.2	±	52.1 abcde	*Tetranychus evansi*, *Tetranychus urticae*	16.0	±	3.8 bc	*Neoseiulus barkeri*, *Amblyseius swirskii*	124.6	±	41.3 a
T7	4	569.8	±	248.1 bcdefg	*Tetranychus evansi*, *Tetranychus urticae*	1.2	±	1.2 a	*Neoseiulus barkeri*	49.0	±	27.7 a
T5	4	1321.0	±	273.6 efg	*Tetranychus evansi*, *Tetranychus urticae*	0.0	±	0.0 abc		72.6	±	12.9 a
S4	4	10844.7	±	985.3 g	*Tetranychus evansi*, Spider mite of the *T. urticae* group	0.0	±	0.0 abc		38.0	±	16.0 a
		*P* < 0.001^1^				*P* < 0.001				*P* < 0.001		

^1^GLM used with a negative binomial distribution. ^2^Different letters indicate a significant difference obtained via pairwise comparison with a Tukey post hoc test

In *Tetranychus* subg. *Tetranychus*, specimens of the two detected non-identified (NI) species were found to belong to the species group N° 9 (Flechtmann and Knihinicki 2002). Among the 38 species that form this group, females have the following characteristics: (i) a hook-shaped peritreme, (ii) diamond-shaped dorsal striation between the third and fourth pairs of dorsocentral setae, (iii) the tarsus of the first leg has four mechanoreceptor setae whose position on the tarsus is proximal to the proximal double setae, and (iv) the empodium has no spur or only a very small one. Characteristics 1, 2 and 4 are common to all species of subg. *Tetranychus*. In this subgenus, the shape and size are key morphological traits for species identification, as the females of the various species are morphologically identical. NI species 1 was sampled on African eggplants in a minimally treated plot in the southern Abidjan area, while NI species 2 was found in the Bassam area in an African eggplant crop plot that had not been treated with chemical pesticides. These two species appear to represent new species; the morphology of their aedeagus and the male empodium of tarsus I are described in Appendices 4 and 5.

Predatory mites of the Phytoseiidae family were found in 11 out of 19 plots in which plant leaves were sampled (Table 1). Five phytoseiid mite species were identified: *Neoseiulus barkeri*, *N. teke*,* Amblyseius swirskii*,* A. tamatavensis* and *Paraphytoseius horrifer*. *A. tamatavensis*,* P. horrifer* and *N. teke* were the majority species found in the southern areas, whereas *N. barkeri* and *A. swirskii* were the predominant species sampled in the northern areas.

Tarsonemid mites were found in 11 out of 19 plots in which plant leaves were sampled (Table 1). Phytophagous *Polyphagotarsonemus latus* was the only tarsonemid species identified but it was always found to be associated with other tarsonemid species but mycophagous not phytophagous.

### Factors affecting the spider mite (*Tetranychus* spp.) abundance

**Fig. 1:**
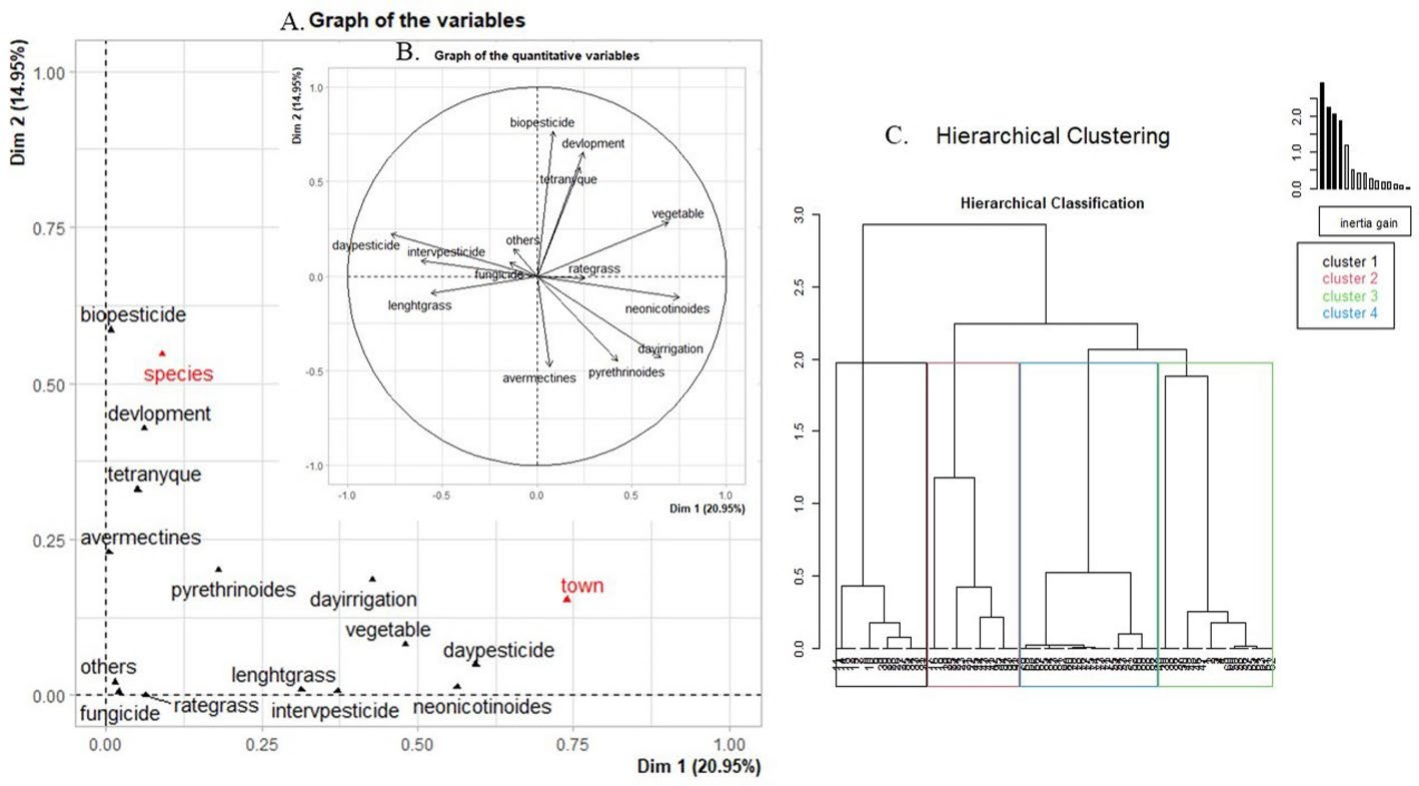
Relationships between agricultural practices and spider mite abundances revealed by FAMD of the variables studied in the factorial space defined by the first two dimensions (Dim 1: 20.95%, Dim 2: 14.95%) and by HCPC. (**A**) Graph of variables: quantitative variables (black) and qualitative variables (red). (**B**) Correlation circle highlighting relationships between the quantitative variables. (**C**) Dendrogram of clusters identified after FAMD

The spider mite abundance rate was positively correlated with the plant growth stage, the use of biopesticides and the extent of crop diversity (Fig. 1), yet it was negatively correlated with the use of pyrethroid and avermectin pesticide treatments. Our hypothesis is that spider mites were more abundant on farms where the crops were at an advanced growth stage, where biopesticides were used and crop diversification was promoted, whereas they were less abundant on farms where pyrethroid and avermectin pesticide treatments were conducted.

HPHC revealed four different clusters (Table 2). The Solanaceae species (*p* < 0.001) and sampling area (*p* < 0.001) had a major impact on the distribution of individuals within the clusters. Neonicotinoid pesticide usage (Eta² = 0.79, *p* < 0.001), the treatment frequency (Eta² = 0.76, *p* < 0.001), date of last treatment (Eta² = 0.65, *p* < 0.001) and weed height (Eta² = 0.61, *p* < 0.001) were the quantitative variables that most contributed to the classification.

**Table 2:** Percentage/average (± SD) of variables according to sampled plot characteristics based on HCPC

Variable	Modality/unit	Cluster 1	Cluster 2	Cluster 3	Cluster 4
Species (%)	Tomato	25.0	**62.5** ^**3**^	**0.0**	**0.0**
	Eggplant	0.0	**0.0**	**91.7**	**11.5**
	African eggplant	75.0	**37.5**	**8.3**	**88.5**
Sampling area (%)	Abidjan	**33.3**	16.7	0.0	**0.0**
	Yamoussoukro	0.0	0.0	**41.7**	0.0
	Bouake	**66.7**	**62.5**	**0.0**	**0.0**
	Korhogo	**0.0**	**20.8**	58.3	**100.0**
Between-treatment interval	N° days	**60.0 ± 0**	**17.3 ± 7.9**	22.7 ± 8.3	23.7 ± 9.8
Time from last treatment	N° days	**50.0 ± 14.1**	17.3 ± 8.3	22.7 ± 8.4	**12.2 ± 8.5**
Pyrethroid treatment	% yes	**8.3**	52.1	**0.0**	**69.2**
Neonicotinoid treatment	%yes	**8.3**	**0.0**	45.5	**100.0**
Avermectin treatment	% yes	25.0	21.7	**0.0**	30.7
Biopesticide usage	% yes	0.0	4.3	**58.3**	**0.0**
Other pesticide treatment	% yes	0.0	**37.5**	**41.6**	**0.0**
Fungicide treatment	% yes	0.0	**20.8**	0.0	0.0
Weed height	cm	**69.2 ± 27.5**	**12.6 ± 8.9**	22.3 ± 17.1	21.9 ± 11.5
Weed cover	%	32.5 ± 24.9	**12.7 ± 1.4**	31.3 ± 27.4	**41.9 ± 22.4**
Time from last watering	Number of days	**1.0 ± 0.0**	2.0 ± 1.4	2.5 ± 0.5	**3.0 ± 0.6**
Crop diversification	% yes	**0.0**	**37.5**	54.5	**100.0**
Crop stage	Level 1 to 4	2.8 ± 0.8	**1.3 ± 1.3**	**3.3 ± 0.5**	**3.0 ± 0.0**
Spider mite	N° spider mites^1^	40.3 ± 11.8a^2^	332.6 ± 186.2b	4377.3 ± 1380.1c	1652.2 ± 684.5c
Phytoseiid mite	N° Phytoseiid mites	1.8 ± 1.5a	4.2 ± 2.2a	8.7 ± 4.3a	4.5 ± 1.5a

^1^*p* < 0.001 (GLM used with a negative binomial distribution); ^2^ Different letters indicate significant differences obtained via pairwise comparison with a Tukey *post hoc* test; ^3^ Numbers in bold indicate that the modality significantly contributed to the classification (*p* < 0.005)

Cluster 1 only contained specimens sampled on tomato and African eggplant crops from the Bouake (*p* = 0.007) and Abidjan (*p* = 0.003) areas (Table 2). A low proportion of spider mites but a higher proportion of predator mites was found. The pesticide treatment frequency was very low (*p* < 0.001) and the last treatment had been conducted a very long time previously (*p* < 0.001). Neonicotinoid (*p* < 0.001) and pyrethroid (*p* < 0.001) pesticides had been seldom used. The weed cover was high (*p* < 0.001). The cropping system was not diversified (*p* < 0.001).

Cluster 2 only contained specimens sampled on tomato (*p* < 0.001) and African eggplant (*p* < 0.001) crops and were mostly from the Bouake (*p* < 0.001) area, with a few from the Korhogo (*p* < 0.001) and Abidjan areas. It was characterized by a moderate spider mite infestation rate with few predator mites present. The cropping system was not very diversified (*p* = 0.021), with a high treatment frequency (*p* < 0.001), particularly with fungicides (*p* < 0.001) and other pesticides (*p* = 0.005). Neonicotinoid pesticides had not been used (*p* < 0.001). The weed height (*p* < 0.001) and weed cover (*p* < 0.001) were low. The crops were at the initial growth stage (*p* < 0.001).

Cluster 3 only contained specimens sampled on eggplant crops (*p* < 0.001) from Yamoussoukro (*p* < 0.001) and Korhogo areas. It was characterized by a high spider mite infestation rate with few predator mites. Avermectin (*p* = 0.048) and pyrethroid (*p* < 0.001) pesticide treatments had not been carried out, but biopesticides (*p* < 0.001) and other pesticides (*p* = 0.003) had been used. The crop growth stage was more advanced (*p* = 0.029) than in the other clusters.

Cluster 4 only contained specimens from the Korhogo (*p* < 0.001) area, that had mainly been sampled on eggplant (*p* < 0.001) crops, with a few on tomato (*p* = 0.012) crops. It was characterized by a high spider mite infestation rate with few predator mites present. The plots had been intensively treated (*p* < 0.001) with neonicotinoid (*p* < 0.001) and pyrethroid (*p* < 0.001) pesticides. Biopesticides (*p* = 0.042) and other pesticides (*p* = 0.042) were not used in this cluster. The weed cover was high (*p* = 0.001). The cropping system was more diversified (*p* < 0.001) and the crop growth stage was more advanced than in the other clusters (*p* = 0.006).

Despite frequent neonicotinoid and pyrethroid pesticide treatments, spider mite infestation rates were very high on eggplant and tomato crops in the Korhogo area (Table 2). Around Bouaké, tomato and African eggplant crop plots were found to have moderate mite infestation rates with intensive pesticide treatments and low weed cover, while tomato and African eggplant crop plots, managed using agroecological practices (scant chemical treatments), had low mite infestation rates, as was also the case in the vicinity of Abidjan. In contrast, in the Yamoussoukro area, eggplant crop plots managed with agroecological practices were heavily infested, especially at the end of the crop season.

### Factors affecting the presence and abundance of spider mites (*Tetranychus* spp.)

**Fig. 2:**
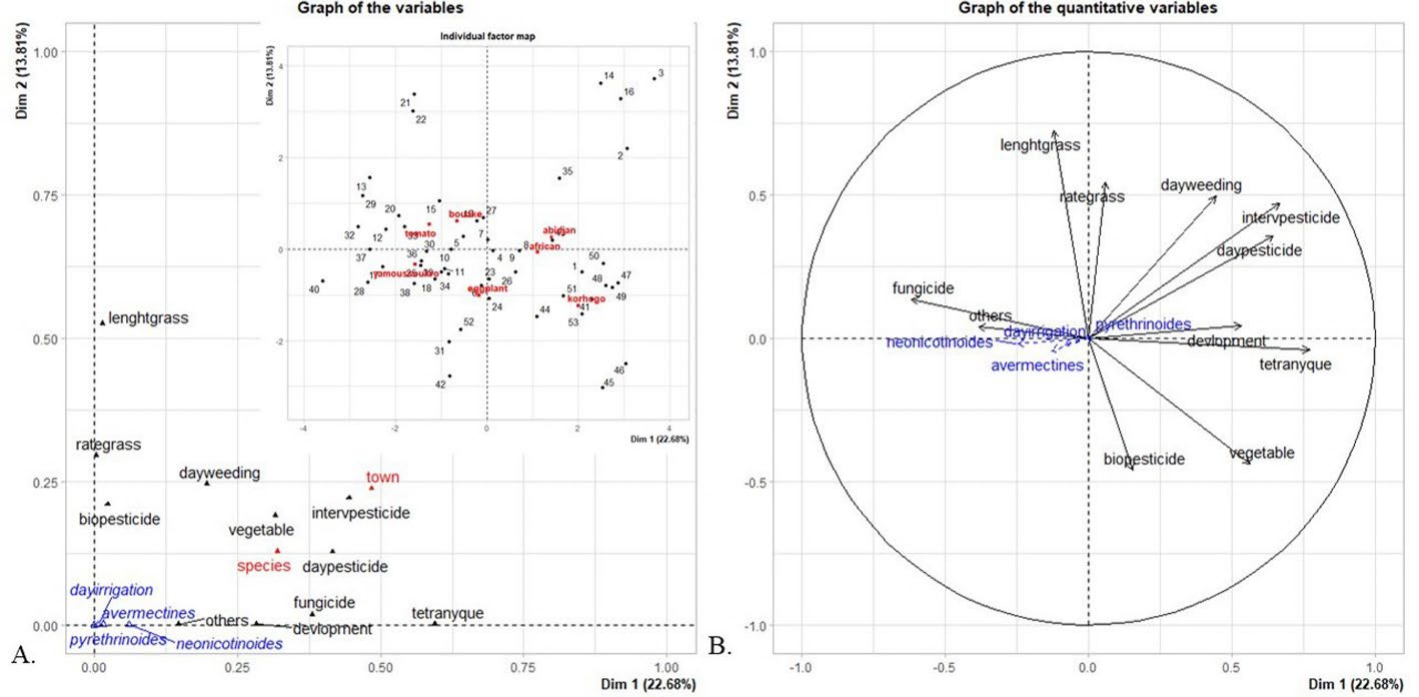
Relationships between agricultural practices and the presence of spider mites illustrated by FAMD of the variables studied in the factorial space defined by the first two dimensions (Dim 1: 22.68%, Dim 2: 13.81%). (A) Graph of variables: quantitative variables (black vectors), qualitative variables (red vectors), and illustrative variables (blue vectors). Individuals (numbered black dots) and qualitative variable modalities (red) are also represented. (B) Correlation circle highlighting relationships between quantitative variables: Projection of quantitative variables (black) and illustrative variables (blue)

Our study of solanaceous crops revealed that 24 of the 53 sampled plots were infested by spider mites (*Tetranychus* spp.). The presence of these pests was positively correlated with the date of the last pesticide treatment, between-treatment interval, weeding date, plant growth stage and crop diversity (Fig. 2). The presence of spider mites seemed to be negatively correlated with fungicide usage. The presence of spider mites was not correlated with: biopesticide usage, the pesticide family, weed height, weed cover, watering date, sampling location or the crop species. Since the last pesticide treatment date and the between-treatment interval were correlated, only the between-treatment interval were accounted for in the statistical analysis.

**Fig. 3:**
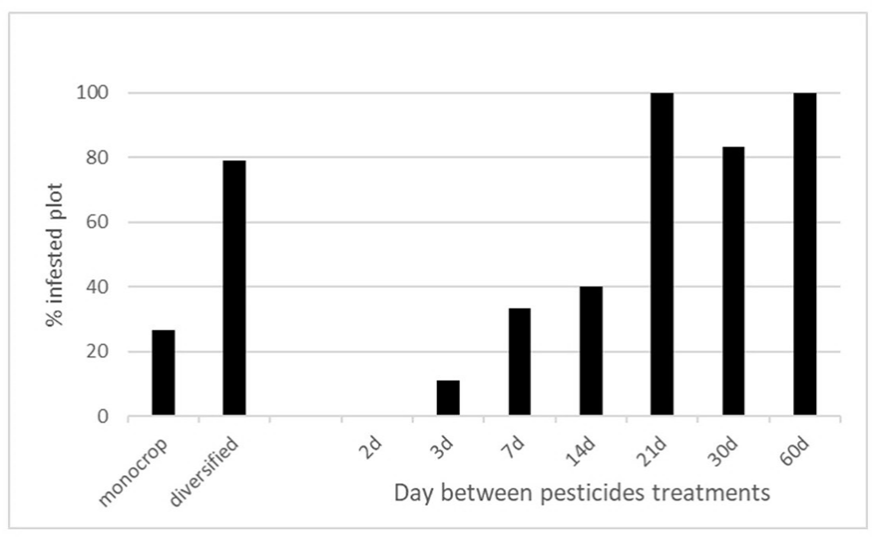
Percentage of spider mite infested plots according to the crop diversity and the pesticide treatment frequency

Our analysis revealed that the crop diversity was conducive to the presence of spider mites (χ^2^ = 15.7, df = 1, *P* < 0.001) and that the number of spider mites decreased as the crop plot pesticide treatment rate increased (χ^2^ = 22.2, df = 1, *P* < 0.001) (Fig. 3). The monitored Solanaceae species, its growth stage, the pesticide chemical family, biopesticide usage, last weeding date, fungicide usage and the sampling area had no impacts on the presence of spider mites.

### Factors affecting the presence and abundance of tarsonid mites

**Fig. 4:**
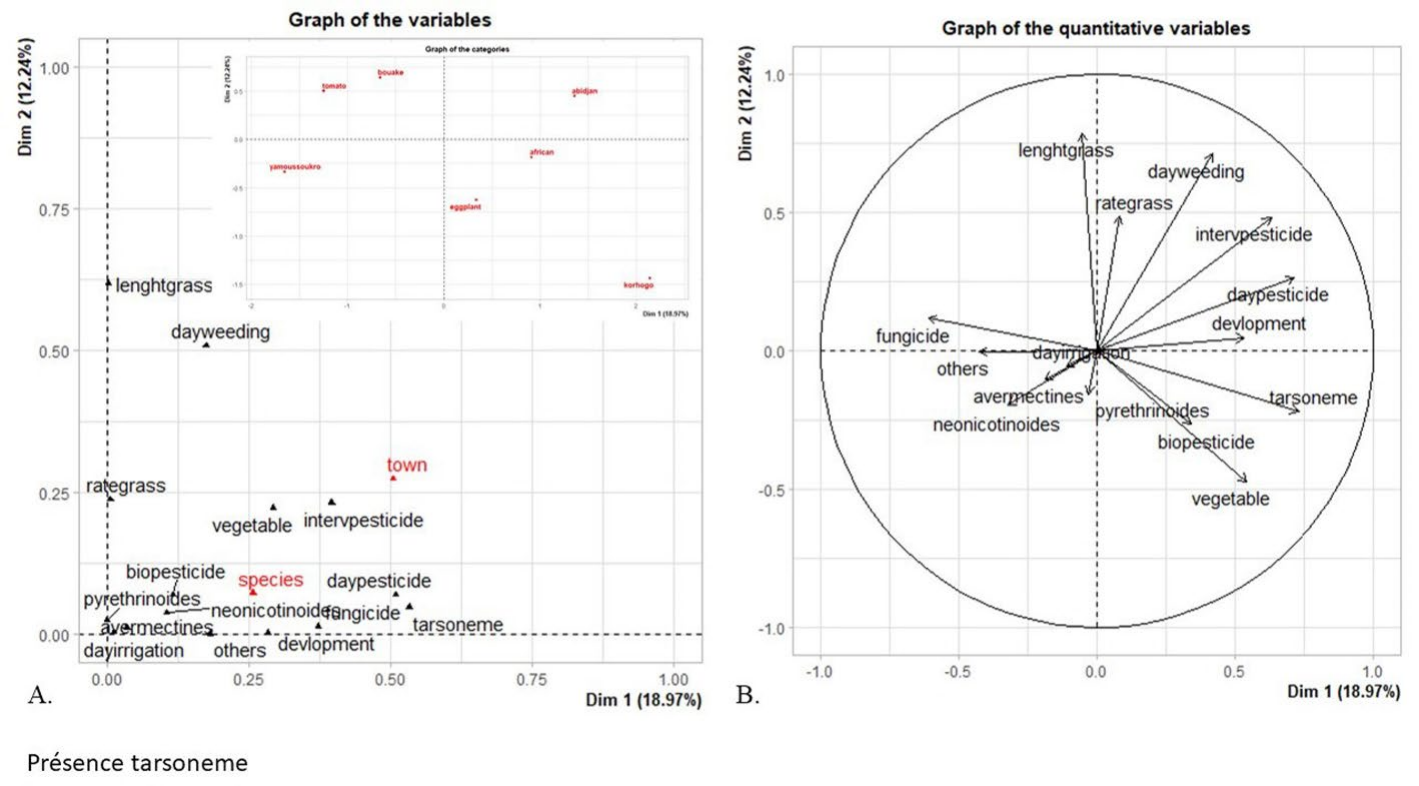
Relationships between agricultural practices and tarsonemid mite abundance rates illustrated by FAMD of the variables studied in the factorial space defined by the first two dimensions (Dim 1: 18.97%, Dim 2: 12.24%). (**A**) Graph of variables: quantitative variables (black) and qualitative variables (red). (**B**) Correlation circle highlighting relationships between the quantitative variables

**Fig. 5:**
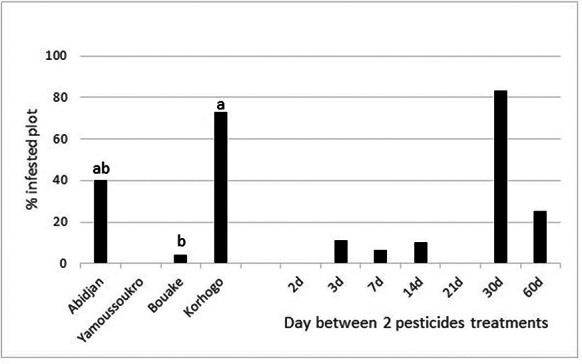
Percentage of tarsonemid infested plots according to the sampling area and the number of days between pesticide treatments (frequency)

^1^GLM used with a binomial distribution, different letters indicate significant differences from pairwise comparison with Tukey *post hoc* test.

FAMD revealed that the presence of tarsonemid mites (*Polyphagotarsonemus latus*) was positively correlated with the crop growth stage, crop diversity, as well as the pesticide treatment date and frequency (Fig. 4). It was also linked to the sampling area and crop type. The presence of tarsonemid mites was negatively correlated with fungicide usage. Our analysis highlighted that the presence of tarsonemid mites was related to the sampling area (χ^2^ = 23.5, df = 3, *P* < 0.001) and treatment frequency (χ^2^ = 6.9, df = 1, *P* = 0.008). Tarsonemid mites were more present in the vicinity of Korhogo in the north than in the Bouake area, and when the treatment frequency was lower (Fig. 5).

Regarding the tarsonemid mite abundance rates, avermectin treatments reduced their abundance (χ^2^ = 49.6, df = 1, *P* < 0.001), as also did the extent of watering, which was often done by sprinkling with a watering can (χ^2^ = 44.5, df = 1, *P* < 0.001).

### Factors affecting the presence and abundance of phytoseiid mites

**Fig. 6:**
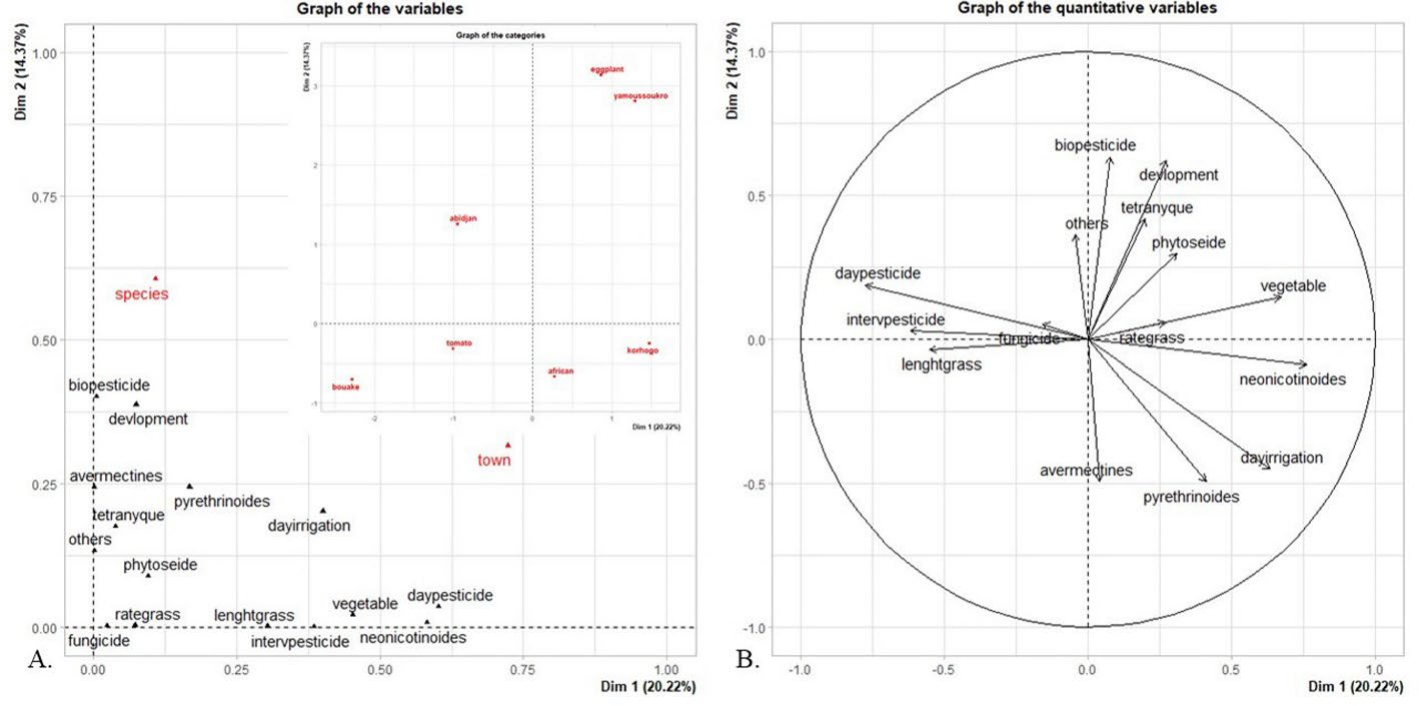
Relationships between agricultural practices and phytoseiid mite abundance rates illustrated by FAMD of the variables studied in the factorial space defined by the first two dimensions (Dim 1: 20.22%, Dim 2: 14.37%) and by HCPC. (**A**) Graph of variables: quantitative variables (black) and qualitative variables (red). (**B**) Correlation circle highlighting relationships between the quantitative variables

FAMD revealed that the presence of phytosiid mites (*Neoseiulus barkeri*, *N. teke*,* Amblyseius swirskii*,* A. tamatavensis* and *Paraphytoseius horrifer*) was positively correlated with the crop growth stage, while the presence of spider mites, crop diversity and biopesticide usage were negatively correlated with the weed height and the pesticide treatment frequency (Fig. 6). Phytoseiid mites were only present in crop plots where spider mites were present (*P* < 0.001), the presence of phytoseiid mites increased with the crop age (*p* = 0.040), when biopesticides were used (*p* = 0.032), when the weed cover was higher (0.023) and when the pesticide treatment frequency decreased (*p* < 0.001).

The phytoseiid mite abundance rate could not be explained by the crop plot type (Table 2) and was not linked to the spider mite abundance (*p* > 0.005). The phytoseiid mite abundance rate was only impacted by the crop growth stage (χ^2^ = 26.8, df = 1, *P* < 0.001)—the phytoseiid mite density increased with the crop age.

### Crop protection practices of the interviewed farmers

11% of the interviewed farmers were using biopesticides. The products used were based on neem oil or extracts from leaves or seeds, black soap, chili pepper extract, garlic, tobacco and kaolin. Kapaas and K-optimal were the two most commonly used pesticides, while 40% of the farmers were using a pesticide mixture (Table 3).

**Table 3:** Pesticides and farmers’ treatment frequency

Brand name	Active ingredient	Pesticide family*	% farmers using this product
Kapaas	Emamectin benzoate	AV	21%
	Abamectin	AV	
	Acetamiprid	NE	
K-optimal	Lambda-cyhalothrin	PY	28%
	Acetamiprid	NE	
Abalone	Abamectin	AV	17%
Ivory	Mancozeb	F	19%
Almanebe	Dithiocarbamate	F	11%
Viper	Acetamiprid	PY	11%
	Indoxacarb	F	
Lambda	Lambda-cyhalothrin	PY	6%
Banko Plus	Chlorothalonil	F	4%
	Carbendazim	F	
Tihan	Spirotetramate	DA	4%
	Flubendiamid	DI	
Kalcuve	Copper oxychloride	F	3%
Cypermax	Cypermethrin	PY	3%
Cypercot	Profenofos	OP	3%
	Cypermethrin	PY	
Hemastar	Emamectin benzoate	AV	2%
K-othrine	Deltamethrin	PY	2%
Fortimec	Abamectin	AV	2%
Biopesticide			11%

*AV: avermectin; NE : neonicotinoid, PY : pyrethroids, OP : organophosphate, F : fungicide DA : tetramic acid derivatives DI : phthalic acid diamides

### Farmers’ knowledge on tomato spider mite pests

91% of the interviewed farmers were unfamiliar with the term ‘*acarien*’ (mite). When shown photographs of plant symptoms, 91% recognised the damage and related it to their own crops. Only 36% linked the symptoms to mites, yet many farmers associated the symptoms with different pest species, e.g. whiteflies or other sucking insects. A majority of the surveyed farmers were unfamiliar with phytophagous mite pests when shown photographs of them, whereas others had nicknames for them such as ‘*petites bêtes*’ (little critters) and ‘*gosounou*’. Farmers who did recognise the damage confirmed that they had observed the onset of symptoms during the dry season, mainly on solanaceous crops.

## Discussion

Our study highlighted the predominance of *T. evansi* spider mites in the four study areas. More than 30% of the monitored plots were infested by *T. evansi*, while 90% were infested by spider mites, with *T. evansi* being the most prevalent species. *T. evansi* has already been identified as an invasive species throughout Africa, with outbreaks of this species first reported in Mauritius in 1960 and then in Zimbabwe in 1982 (Boubou et al. 2011). This mite has since become a major pest of solanaceous crops, especially in Benin, where it has replaced *T. urticae* and *T. ludeni*, the only spider mite species identified in the early 2000 s (Adango et al. 2006; Azandémè-Hounmalon et al., 2015). Our survey in Côte d’Ivoire revealed that phytoseiid mites were either very rare or present in quantities that were too low to keep spider mite populations in check, even in pesticide untreated plots. This absence of local predators could explain the observed *T. evansi* outbreaks. A previous survey conducted by Azandeme Hounmalon (2015) in southern Benin also highlighted the absence of natural enemies in a *T. evansi* outbreak area. Otherwise, Adango et al. (2006) conducted a study prior to any *T. evansi* outbreaks in Benin and detected the presence of local natural enemies, i.e. mainly *Iphiseius degenerans* (Berlese) and *Ueckermannseius saltus* (Denmark & Matthysse) in untreated plots. The absence of natural enemies was not the sole explanation for the *T. evansi* abundance rates noted in our study. Although we did not obtain any evidence of this in our study, extensive pesticide use may have wiped out a large proportion of the phytoseiid mite populations (Zahid et al. 2017), thereby indirectly promoting outbreaks of certain phytophagous mite species. *T. evansi* also has a greater development rate than *T. urticae*, which may have enabled it to proliferate in the area (Bonato 1999; Migeon 2005). *T. evansi* identification is essential to be able tailor recommended measures for controlling this pest to benefit farmers in Côte d’Ivoire. Unlike *T. urticae*,* T. evansi* is a gregarious spider mite species that develops on a single plant and—once a certain population density is reached—this pest can spread throughout the crop plot (Azandémè-Hounmalon et al. 2014). The high variability in our counts of this species confirmed this gregarious behavioural trend, with the presence of infestation hotspots. The latter could be detected early, i.e. upon the onset of infestation symptoms, and controlled before the infestation spreads throughout the plot.

Although *T. evansi* was the most common species, it was not the only phytophagous mite species found. Indeed, *T. urticae* was identified on a few plots in the Korhogo area, and unidentified species of the *T. urticae* group were noted in all four locations. Moreover, it was astonishing that we did not identify any *T. ludeni* mites, which have been reported in Benin (Adango et al. 2006), or *T. neocaledonicus mites*, which are very common in intertropical regions and had been detected in Côte d’Ivoire in 1985 and 1986 (Migeon 2021). Note, however, that these samples had been collected one or more decades prior to our study, so these species are likely no longer major elements in the current phytophagous mite diversity pattern in the country. Also, Da-Costa et al. (2025) showed *T. ludeni* has a low population growth in plants with *T. urticae*. More recent surveys carried out by Azandémè-Hounmalon et al. (2018b) in southern Benin did not identify any species other than *T. evansi*. This species appears to have overtaken other local spider mite species e.g. *T. ludeni* which, if present, now seem to be very few in number. Two unidentified spider mite species were discovered in the vicinity of Abidjan, whose presence could possibly be explained by their short life cycle, which may have facilitated the development of resistance to certain pesticide active ingredients or families (Cranham and Helle 1985). This might have enabled them to thrive in this area despite the frequent pesticide treatments. Periurban Abidjan appeared to be the area with the greatest overall mite species diversity, even though the spider mite abundance rates were substantially lower.

Spider mites were found in all four study areas but were more prevalent around Korhogo. This area had a savanna-like landscape, so the climatic conditions were drier than in the other study areas, and it is known that spider mites thrive mainly when temperatures are high with low relative humidity (De Moraes and McMurtry 1987). During the sampling period, the mean temperature was 29 ± 8 °C with 58% relative humidity (meteoblue, 2022), which are ideal conditions for spider mites (Bonato 1999; Kumral et al. 2019). In contrast, as tarsonemid mites thrive in relatively humid environments (Blancard and Ryckewaert 2021), the climatic conditions in the Korhogo area would not seem very compatible for these mites. Yet tarsonemid mites were only detected in this specific area, i.e. in lowlands where rainfed rice was cropped part of the year. During the sampling period, most of the plants in this location were partly submerged in water due to the proximity of a lake. Spider mite numbers were lower in these plots than in the other plots sampled in the Korhogo area.

Phytophagous mites and their natural enemies may be affected by diverse agroecological conditions, such as the host plant (species and variety), temperature, relative humidity, light, surrounding vegetation diversity and the nutritional quality of the infested plants (Azandémè-Hounmalon et al. 2014). Agricultural practices also impact the dynamics of pest populations and those of their natural enemies. Our study aimed to characterise the diversity and abundance of solanaceous crop pest mites in Côte d’Ivoire and their natural enemies, while assessing the extent to which agricultural practices affect these pests. Our hypothesis was that frequent pesticide use or heavy weed cover would reduce the presence of spider mites, while the crop diversity and growth stage would increase it. Based on interview, we highlighted that farmers did not know much about mites and how to control them. It would hence be essential to raise their awareness regarding mite infestations and disseminate information on suitable control methods to maximize the effectiveness of their crop protection strategies. The survey confirmed that spider mite control by farmers was almost entirely based on chemical pesticide treatments, often applied randomly, without sufficient control or protective equipment. These findings corroborate those obtained in recent surveys conducted in the Yamoussoukro region (Diabaté et al. 2022) and in Benin (Azandémè-Hounmalon et al. 2018b). Crop protection products that are often not approved for use on vegetable crops, but rather for cash crops such as cotton, were commonly used (Tarnagda et al. 2017; De Bon et al. 2019).

The interview surveys highlighted the widespread use of pesticides, particularly the simultaneous application of mixtures of commercial products by 40% of farmers. The pesticides most commonly used, e.g. Kapaas and K-optimal, contained several active ingredients. The most frequently treated plots had fewer mites, but the extent of infestation did not seem to be related to the treatment frequency. The less frequently treated plots in the vicinity of Abidjan and Bouaké had fewer mites than the highly treated plots around Bouaké and Korhogo. Untreated plots in the Yamoussoukro area were heavily infested by mites, as were the highly treated plots around Korhogo. The pesticide chemical families might have had an impact. Farmers who were generally not applying pyrethroid and neonicotinoid pesticides seemed to have fewer mite outbreaks than those who applied high quantities of biopesticides or neonicotinoids. It would, however, be hard to attribute the effects of a pesticide family to the mite infestation levels because of the active ingredient mixtures in some pesticide products, and the homemade pesticide blends that some farmers applied. Some interviewed farmers claimed that only abamectin was effective in controlling spider mites, unlike other pesticides used in market gardening systems. However, it has been reported that uncontrolled repeat use of pesticides tends to enhance the spread of spider mite populations since their main natural enemies—phytoseiid mites—are generally highly susceptible to pesticides (Adango et al. 2006). Several studies have shown that avermectin and pyrethroid pesticides can generally wipe out most of the population of the main spider mite species, although pyrethroid resistance is common (Lagziri et al. 2015; El-Tahawe and Abd El-Rahman H 2017). Only avermectin and organophosphate pesticides have shown some degree of efficacy in controlling *P. latus* (Etienne et al. 2020), whereas pyrethroids do not seem effective for controlling this tarsonemid mite (Vaissayre 1986; Renou and Chenet 1988). The development of populations resistant to certain active ingredients or even pesticide families is common when they have been applied excessively, as has been reported regarding some *T. evansi* populations in Kenya (Toroitich 2006; Toroitich et al. 2014). Some fungicides such as sulphur (Auger et al. 1999) seem to be able to curb *T. urticae* infestations but these treatments also sometimes reduce phytoseiid mite populations (Kreiter et al. 1998; Alston and Thomson 2004). In addition, a study in southern Benin carried out by Adango et al. (2006) highlighted the presence of a low number of natural enemies on treated plots compared to untreated plots. This clearly shows that some pesticides, particularly pyrethroids, have a detrimental effect on agrosystem biodiversity (Lee et al. 2002; Ministère de l’agriculture et de l’agroalimentaire, 2017; Cheng et al. 2018). It would be interesting to assess the extent of spider mite resistance to the various insecticides commonly used by farmers. *T. evansi* and *T. urticae* are known to be resistant to various pesticides (Nyoni et al. 2011; Zhang et al. 2022), but little data is available for West Africa (Eziah et al. 2016). Alternative products that are less toxic to natural enemies are now being recommended, including neem oil and extracts from plants such as chili pepper and garlic (Schmutterer et al. 1983; Fatima et al. 2015). In the present study, biopesticides only seemed effective for a short period of time. High spider mite infestations were noted at the end of the crop cycle in agroecological plots monitored in the vicinity of Yamoussoukro (cluster 3).

Phytoseiid mite densities were too low to provide effective natural control of spider mite or tarsonemid mite infestations on solanaceous crops in Côte d’Ivoire. Our results highlight the need to promote conservation biological control strategies based on the preservation and enhancement of local predatory mite communities. Some of the identified species have been described as potential natural enemies of certain phytophagous mite families and species. *A. swirskii*, for instance, was shown to be an effective *T. evansi* and *P. latus*. Indigenous phytoseiid mites may play a key role in regulating populations of *T. evansi* and *T. urticae* when suitable habitats and alternative resources are maintained. However, the effectiveness of this predator is curbed by the presence of glandular trichomes on solanaceous plants (Houten et al. 2013; Paspati 2019). Otherwise, *N. barkeri* may be worth preserving in cropfields as it generally feeds on *P. latus* and may also prey on *T. urticae* when no other food source is available (Bonde 1989). In addition, augmentative releases of the specialist predator *Phytoseiulus persimilis* could be considered as a complementary control strategy, particularly during periods of high pest pressure. The combined use of conservation and augmentative biological control approaches may offer a sustainable and effective alternative to intensive chemical treatments for managing *T. evansi* in solanaceous cropping systems. The predatory species *P. longipes* has proven to be highly effective against *T. evansi*, suggesting that it could be introduced in Côte d’Ivoire to curtail infestations of this phytophagous mite (Ferrero 2006; Ferrero et al. 2007; Azandeme Hounmalon 2015). This predator specifically targets *T. evansi* spider mites and adapts well to solanaceous crops, indicating that it should be suitable for use in biocontrol programmes. Its resistance to high temperatures and drought is also an advantage over other phytoseiid mite species (Ferrero et al. 2007). Studies are underway in Benin to assess its predation potential and adaptation to local conditions (Azandeme, personal communication). The various specimens sampled in the four areas of Côte d’Ivoire revealed that there was greater phytoseiid mite diversity in the Abidjan area. This could be explained by the fact that the climatic conditions were conducive to the development of these predatory mites, which prefer high relative humidity and milder temperatures, i.e. at > 30 °C the conditions are no longer suitable for some species (Skirvin and Fenlon 2003; De Courcy Williams et al. 2004; Ferrero et al. 2010). *A. tamatavensis* was only found in the Abidjan area. It was surprising that no mites of this species were collected in Korhogo, where the plots were surrounded by banana trees, despite the fact that this species has been noted on *Musa paradisiaca* crops in West Africa (Demite et al. 2022). The high temperatures in northern Côte d’Ivoire, particularly in April and May (i.e. our sampling period), as well as the heavy pesticide treatments, might explain their absence. The diversity noted in the Korhogo area encompassed three mite species. *A. tamatavensis* mites were only found in the Natiokobadara area, where vegetables were being cropped in a humid area near a water source in rice fields. The humid conditions and the breadth of the host plant leaves (African eggplant), which provided sufficient shade for them, could explain their presence.

## Conclusions

This scoping study conducted in four areas of Côte d’Ivoire involved sampling in 53 tomato, eggplant and African eggplant plots. More than 30% of the studied plots were infested by *T. evansi*, which was the dominant species in 90% of the mite-infested plots. *T. urticae* and *P. latus* were also identified, along with two unidentified species and species of the *T. urticae* group. Several natural enemies were identified, including *Neoseiulus barkeri*, *N. teke*,* Amblyseius swirskii*,* A. tamatavensis* and *Paraphytoseius horrifer*, but their abundance was too low to enable effective natural control. Spider mite infestation rates decreased as the pesticide treatment frequency increased. Mite infestation levels were higher in areas with greater crop diversity. Spider mites were present in all four study areas but were more prevalent in the vicinity of Korhogo. The mite diversity was nevertheless greater in the Abidjan area. The farmer interviews highlighted their extensive use of pesticides, particularly the simultaneous application of commercial pesticide mixtures by 40% of the farmers. The most commonly used products, i.e. Kapaas and K-optimal, are pesticides containing several active ingredients. The most heavily treated plots were less affected by mites, but the infestation rates did not appear to be related to the treatment frequency. The lack of knowledge about mites and effective control methods appeared to be hampering phytophagous mite control and reducing chemical pesticide usage. One solution could be to introduce an effective predator such as *Phytoseiulus longipes.*

## Supplementary Information

Below is the link to the electronic supplementary material.

Supplementary Material 1

Supplementary Material 2

Supplementary Material 3

Supplementary Material 4

Supplementary Material 5

## Data Availability

Data will be made available on request.

## References

[CR1] Adango E, Onzo A, Hanna R, Atachi P, James B (2006) Inventaire de la faune des acariens sur Amaranthus cruentus (Amaranthaceae), Solanum macrocarpon et Solanum aethiopicum (Solanaceae) dans le Sud Benin. Int J Trop Sci 26(3):155–165

[CR2] Adango E, Onzo A, Hanna R, Atachi P, James B (2007) Mite pests of major importance on indigenous leafy vegetables in Benin: the search for appropriate control strategies. Acta Hortic 752:311–317

[CR3] Adango E, Onzo A, Padonou UAVL (2020) Diversité des espèces de cultures maraîchères et d’adventices hôtes de l’acarien Tarsoneme Polyphagotarsonemus latus Banks (Acari: Tarsonemidae) sur quelques périmètres maraîchers du Sud-Bénin. J Appl Biosci 149:15310–15321

[CR4] Adetonah S, Koffi-Tessio E, Coulibaly O, Eric S, Mensah G (2011) Perceptions et adoption des méthodes alternatives de lutte contre les insectes des cultures maraichères en zone urbaine et péri urbaine au Bénin et au Ghana. Bulletin de la Recherche Agronomique du Bénin 69:1–10

[CR5] Alston DG, Thomson SV (2004) Effects of fungicide residues on the survival, fecundity, and predation of the mites *Tetranychus urticae* (Acari: Tetranychidae) and *Galendromus occidentalis* (Acari: Phytoseiidae). J Econ Entomol 97(3):7 10.1093/jee/97.3.950 15279277

[CR6] Auger P, Tixier MS, Kreiter S, Vergonjeanne H (1999) Grapevine. The miticide effects of sulphur in the control of *Tetranychus urticae* spider mite. Phytoma 515:24–29

[CR7] Azandeme Hounmalon YG (2015) Comportement de Tetranychus evansi sur tomate et interaction avec son prédateur Phytoseiulus longipes. Application pour une stratégie de lutte intégrée en condition tropicale. Montpellier SupAgro, thesis

[CR8] Azandémè-Hounmalon GY, Fellous S, Kreiter S, Fiaboe KKM, Subramanian S, Kungu M, Martin T (2014) Dispersal behavior of *Tetranychus evansi* and T. urticae on tomato at several spatial scales and densities: implications for integrated pest management. PLoS One 9(4):e95071 24743580 10.1371/journal.pone.0095071 PMC3990603

[CR9] Azandémè Hounmalon GY, Maniania NK, Niassy S, Fellous S, Kreiter S, Delétré E, Fiaboe KK, Martin T (2018a) Performance of *Metarhizium anisopliae*-treated foam in combination with *Phytoseiulus longipes* Evans against *Tetranychus evansi* Baker & Pritchard (Acari: Tetranychidae). Pest Manag Sci 74(12):2835–2841 29756384 10.1002/ps.5073

[CR10] Azandémè-Hounmalon YG, Affognon HD, Assogba Komlan F, Tamo M, Fiaboe KKM, Kreiter S, Martin T (2018b) Farmers’ perception and control practices against the invasive red spider mite (Tetranychus evansi Baker and Pritchard) in Benin. Proceedings of the III All Africa Horticultural Congress, 8

[CR11] Azandémè-Hounmalon GY, Sikirou R, Onzo A, Fiaboe KKM, Tamò M, Kreiter S, Martin T (2022) Re-assessing the pest status of *Tetranychus evansi* (Acari: Tetranychidae) on solanaceous crops and farmers control practices in Benin. J Agric Food Res. 10.1016/j.jafr.2022.100401

[CR12] Blancard D, Ryckewaert P (2021) Polyphagotarsonemus latus (Banks) Tarsoneme - acariose déformante. E-Phytia. [consulté le 24 mai 2022]. [http://ephytia.inra.fr/fr/C/24347/Tropileg-Acariose-deformante-tarsoneme](http://ephytia.inra.fr/fr/C/24347/Tropileg-Acariose-deformante-tarsoneme)

[CR13] Bonato O (1999) The effect of temperature on life history parameters of *Tetranychus evansi* (Acari: Tetranychidae). Exp Appl Acarol 23(1):11–19

[CR14] Bonde J (1989) Biological studies including population growth parameters of the predatory mite *Amblyseius barkeri* [Acarina.: Phytoseiidae] at 25°C in the laboratory. Entomophaga 34(2):275–287

[CR15] Boubou A, Migeon A, Roderick GK, Navajas M (2011) Recent emergence and worldwide spread of the red tomato spider mite, Tetranychus evansi: genetic variation and multiple cryptic invasions. Biol Invasions 13(1):81–92

[CR16] Cheng S, Lin R, Zhang N, Yuan S, Zhou X, Huang J, Ren X, Wang S, Jiang H, Yu C (2018) Toxicity of six insecticides to predatory mite Amblyseius cucumeris (Oudemans) (Acari: Phytoseiidae) in- and off-field. Ecotoxicol Environ Saf 161:715–720 29940512 10.1016/j.ecoenv.2018.06.018

[CR17] Cranham JE, Helle W (1985) Pesticide Resistance in Tetranychidae. In: Spider mites, their biology, natural enemies and control. Amsterdam: pp. 405–421 (Coll. World Crop Pests)

[CR18] Da-Costa T, Dos Santos CF, Rodighero LF, Ferla NJ, Soares GLG (2025) Interaction between Tetranychus ludeni and Tetranychus urticae (Acari: Tetranychidae) on bean plants affects their biological performance. Exp Appl Acarol 95(3):32 40839024 10.1007/s10493-025-01052-4

[CR19] De Bon H, Fondio L, Dugué P, Coulibali Z, Biard Y (2019) Etude d’identification et analyse des contraintes à la production maraîchère selon les grandes zones agro-climatiques de la Côte d’Ivoire. Cirad, Côte d’Ivoire, p 140

[CR20] De Courcy Williams ME, Kravar-garde L, Fenlon JS, Sunderland KD (2004) Phytoseiid mites in protected crops: the effect of humidity and food availability on egg hatch and adult life span of *Iphiseius degenerans*, *Neoseiulus cucumeris*, *N. californicus* and *Phytoseiulus persimilis* (Acari: Phytoseiidae). Exp Appl Acarol 32(1):1 15139268 10.1023/b:appa.0000018170.46836.11

[CR21] De Moraes GJ, McMurtry JA (1987) Effect of temperature and sperm supply on the reproductive potential of Tetranychus evansi (Acari: Tetranychidae). Exp Appl Acarol 3(2):95–107

[CR22] Demite PR, McMurtry JA, Moraes GJ (2022) Phytoseiidae Database. Esalq. [consulté le 24 mai 2022]. http://www.lea.esalq.usp.br/phytoseiidae/

[CR23] Diabate S, Tchicaya E, Pabo QO, Belmin R, Djezou B, Ahouangninou L, Fondio L, Kone D, Martin T (2022) A bio-test to assess the risk of chemical insecticide residues in soil and. vegetable crops pas de nom de journal

[CR24] Drabo E, Sanou A, Boly A, OUura A, Waongo A, Traore F, Ilboudo Z, Dabire-Binso LC, Sanon A (2023) Criblage de cinq variétés de tomate contre Tetranychus evansi Baker and Pritchard, 1960 (Acari: Tetranychidae), acarien ravageur de la tomate en culture au Burkina Faso. Sci Nat Et Appliquées 41(2):239–251. https://revuesciences-techniquesburkina.org/index.php/sciences_naturelles_et_appliquee/article/view/1150

[CR25] El-Tahawe HS, Abd El-Rahman HHA (2017) Effect of temperature and lambda-cyhalothrin on development of immature stages of the two spotted spider mite, Tetranychus urticae Koh. Zagazig J Agricultural Res 44(6):2695–2702

[CR26] Etienne A, Alexis O, Wilson DCOG (2020) Evaluation de L’activité Acaricide de quelques biopesticides sur l’acarien Tarsoneme, Polyphagotarsonemus latus Banks (Acari: Tarsonemidae) infestant l’aubergine Gboma (Solanum macrocarpon L.) au Sud-Bénin. European Scientific Journal ESJ. 10.19044/esj.2020.v16n15p442

[CR27] Eziah VY, Buba RB, Afreh-Nuamah K (2016) Susceptibility of two spotted spider mite Tetranychus urticae KOCH (Acari; Tetranychidae) to some selected miticides in the Greater Accra Region of Ghana. Int J Biol Chem Sci 10(4):1473–1483

[CR28] Fan Y, Petitt FL (1994) Functional response of Neoseiulus barkeri Hughes on two-spotted spider mite (Acari: Tetranychidae). Exp Appl Acarol 18(10):613–621. 10.1007/BF00051724

[CR29] Fatima K, Lovejoy T, Wisdom K (2015) Efficacy of Garlic (Allium sativum) and Red Chilli Pepper (Capsicum annum) Extracts in the Control of Red Spider Mite (Tetranychus urticae) in Tomatoes (Lycopersicon esculentum). Asian J Appl Sci 3(1):124–131

[CR30] Fauvel G, Cotton D (1983) Application de la technique du lavage au dénombrement des oeufs d’hiver de l’acarien rouge *Panonychus ulmi* Koch (Acari, Tetranychidae). Agronomie 3(5):483–486

[CR31] Ferrero M (2006) Le système tritrophique tomate-Tetranyques tisserands-Phytoseiulus longipes - Etude de la variabilité des comportements alimentaires du prédateur et conséquences pour la lutte biologique. Montpellier SupAgro, Montpellier, p 237

[CR32] Ferrero M, de Moraes GJ, Kreiter S, Tixier MS, Knapp M (2007) Life tables of the predatory mite Phytoseiulus longipes feeding on Tetranychus evansi at four temperatures (Acari: Phytoseiidae, Tetranychidae). Exp Appl Acarol 41(1):45 17334816 10.1007/s10493-007-9053-6

[CR33] Ferrero M, Gigot C, Tixier M-S, Van Houten YM, Kreiter S (2010) Egg hatching response to a range of air humidities for six species of predatory mites. Entomol Exp Appl 135(3):237–244

[CR34] Flechtmann CH, Knihinicki DK (2002) New species and new record of Tetranychus Dufour from Australia, with a key to the major groups in this genus based on females (Acari: Prostigmata: Tetranychidae). Aust J Entomol 41(2):118–127

[CR35] Gutierrez J (1989) Les acariens phytophages des principales cultures tropicales. Colloques sur les acariens des cultures. INRA-ORSTOM, France, p 10

[CR36] INRAe (2018) Hypp: encyclopédie en protection des plantes - Acariens (Acari). Ephytia. [consulté le 28 février 2022]. http://ephytia.inra.fr/fr/C/11094/Hypp-encyclopedie-en-protection-des-plantes-Acariens-Acari

[CR37] Krantz GW, Walter DE (2009) A manual of acarology

[CR38] Kreiter S, Sentenac G, Weber M, Rinville C, Auger P (1998) Effets non intentionnels de quelques produits phytopharmaceutiques sur Typhlodromus pyri, Kampidromus aberrans et Phytoseius plumifer. Phytoma 505:51–58

[CR39] Kumral NA, Göksel PH, Aysan E, Kolcu A (2019) Life table of *Tetranychus urticae* (Koch) (Acari: Tetranycidae) on different Turkish eggplant cultivars under controlled conditions. Acarologia 59(1):12–20

[CR40] Kungu M, Deletre E, Subramanian S, Fiaboe K, Gitonga L, Osiemo Lagat Z, Martin T (2018) A new mite IPM strategy: predator avoidance behaviour resulting from the synergetic effects of predator release and acaricide-treated nets. Pest Manag Sci. 10.1002/ps.5203 30203617 10.1002/ps.5203

[CR41] Kungu M, Subramanian S, Salifu D, Fiaboe K, Azandémè-Hounmalon GY, Gitonga L, Onyambu GK, Deletre E, Martin T (2020) Influence of Predatory Mites, Phytoseiulus longipes Evans, on the Within-Plant Diurnal Migration and Distribution of the Red Spider Mite, Tetranychus evansi, Baker and Pritchard on African Nightshade, Solanum scabrum. In: Niassy S, Ekesi S, Migiro L, Otieno W (eds) Sustainable Management of Invasive Pests in Africa. Sustainability in Plant and Crop Protection, vol 14. Springer, Cham, pp 267–282. 10.1007/978-3-030-41083-4_21

[CR42] Lagziri M, Benicha M, M’rabet R, Amrani AE (2015) Influence de l’usage préventif des pesticides sur les acariens Tetranychus urticae et Phytoseiulus persimilis (Acari: Tetranychidae, Phytoseiidae) présents en cultures de fraisiers du Nord du Maroc. Biotechnol Agron Soc Environ, 9

[CR43] Lee SG, Hilton SA, Broadbent AB, Kim JH (2002) Insecticide resistance in Phytoseiid predatory mites, *Phytoseiulus persimilis* and *Amblyseius cucumeris* (Acarina: Phytoseiidae). J Asia Pac Entomol 5(1):123–129

[CR44] meteoblue (2022) Weather Archive Korhogo. meteoblue. [consulté le 13 septembre 2022]. https://www.meteoblue.com/en/weather/historyclimate/weatherarchive/korhogo_ivory-coast_2286304

[CR45] Migeon A (2005) Un nouvel acarien ravageur en France: Tetranychus evansi Baker & Pritchard. Phytoma - La Défense des Végétaux 579:38–43

[CR46] Migeon A (2021) Spider Mites Collection of Jean Gutierrez. Gbif. [consulté le 16 mars 2022]. https://www.gbif.org/dataset/ac60a288-fcc9-43fe-a7d4-e732b748a981

[CR47] Migeon A, Dorkeld F (2022) Spider Mites Web: a comprehensive database for the Tetranychidae. Spider Mites Web. [consulté le 13 septembre 2022]. http://www1.montpellier.inra.fr/CBGP/spmweb/

[CR48] Ministère de l’agriculture et de l’agroalimentaire (2017) Le catalogue des effets non intentionnels des produits phytosanitaires. E-Phy. [consulté le 25 mai 2022]. http://e-phy.agriculture.gouv.fr/

[CR49] Momen FM, Elsaway SA (1993) Biology and feeding behavior of the predatory mite, *Amblyseius swirskii* (Acari, Phytoseiidae). Acarologia 34(3):199–204

[CR50] Moraes G, Zannou I, Ueckermann E, Oliveira A, Yaninek J, Hanna R (2007) Phytoseiid mites of the tribes Afroseiulini, Kampimodromini and Phytoseiulini, and complementary notes on mites of the tribes Euseiini and Neoseiulini (Acari: Phytoseiidae) from sub-Saharan Africa. Zootaxa. 10.11646/zootaxa.1628.1.1

[CR51] Nyoni BN, Gorman K, Mzilahowa T, Williamson MS, Navajas M, Field LM, Bass C (2011) Pyrethroid resistance in the tomato red spider mite, *Tetranychus evansi*, is associated with mutation of the para-type sodium channel. Pest Manag Sci 67(8):891–897 21432985 10.1002/ps.2145

[CR52] Okoth SA, Okoth P, Wachira PM, Roimen H (2009) Spatial distribution of trichoderma spp. in embu and taita regions, Kenya. Tropical and Subtropical Agroecosystems, p 13

[CR53] Onzo A, GAR Tossounon Yarou (2015) Inventaire des acariens et insectes ravageurs associés à la culture du piment vert Capsicum chinense Jacq. (Solanales: Solanaceae) dans les communes de Kandi et Malanville au Nord-Bénin. 5(1):1–11

[CR54] Onzo A, Houedokoho AF, Hanna R (2012) Potential of the predatory mite, *Amblyseius swirskii* to suppress the broad mite, *Polyphagotarsonemus latus* on the gboma eggplant, *Solanum macrocarpon*. J Insect Sci 12(1):7 22962997 10.1673/031.012.0701 PMC3465922

[CR55] Paspati A (2019) Living on an unfriendly plant host: impact of tomato on the predatory mite Amblyseius swirskii. Universitat Jaume I, PhD, Castelló de la Plana

[CR56] Pritchard AE, Baker EW (1955) A revision of the spider mite - Family Tetranychidae. The Pacific coast entomological society. Etats-Unis: 472 p

[CR57] Renou A, Chenet T (1988) Efficacité de la biphenthrine en culture cotonière au Nord-Cameroun. Cot Fib Trop 43(3):227–233

[CR100] Sahraoui H (2012) Influence des pratiques agro-écologiques et de la protectionphytosanitaire sur les communautés d’acariens Phytoseiidae (Acari mesostigmata) dans lesvergers agrumicoles tunisiens. Montpellier, France : SupAgro, 198 p.

[CR58] Saunyama IGM, Knapp M (2003) Effect of pruning and trellising of tomatoes on red spider mite incidence and crop yield in Zimbabwe. Afr Crop Sci J 11(4):269–277

[CR59] Schmutterer H, Deutsche GfuerT, Zusammenarbeit E, Rauischholzhausen INC, Ascher KRS (1983, 1984) Natural pesticides from the neem tree (Azadirachta indica A. Juss) and other tropical plants. Proceedings of the 2nd Internat Neem Conference Rauischholzhausen. TZ-Verlagsgesellschaft. Allemagne: Deutsche Gesellschaft fuer Technische Zusammenarbeit, 587 p. (Coll. International Neem Conference)

[CR60] Skirvin DJ, Fenlon JS (2003) The effect of temperature on the functional response of *Phytoseiulus persimilis* (Acari: Phytoseiidae). Exp Appl Acarol 31(1):37 14756399 10.1023/b:appa.0000005107.97373.87

[CR61] Song ZW, Zheng Y, Zhang BX, Li DS (2016) Prey consumption and functional response of *Neoseiulus californicus* and *Neoseiulus longispinosus* (Acari: Phytoseiidae) on *Tetranychus urticae* and *Tetranychus kanzawai* (Acari: Tetranychidae). Syst Appl Acarology 21(7):936–946

[CR62] Tarnagda B, Tankoano A, Tapsoba F, Sourabié Pane B, Abdoullahi Hissein O, Oumar Djbrine A, Maxim Drabo K, Traoré Y, Aly S (2017) Évaluation des pratiques agricoles des légumes feuilles: le cas des utilisations des pesticides et des intrants chimiques sur les sites maraîchers de Ouagadougou, Burkina Faso. J Appl Biosci 117:11. 10.4314/jab.v117i1.3

[CR63] Toroitich FJ (2006) Effect of pesticides on the tobacco spider mite Tetranychus evansi Baker & Pritchard on tomatoes in Kenya. University Of Nairobi, Kenya, p 69

[CR64] Toroitich FJ, Knapp M, Nderitu JH, Olubayo FM, Obonyo M (2014) Susceptibility of geographically isolated populations of the Tomato red spider mite (*Tetranychus evansi* Baker & Pritchard) to commonly used acaricides on tomato crops in Kenya. J Entomol Acarological Res 46(1):18–25

[CR65] Vaissayre M (1986) Lutte chimique contre l’acarien Polyphagotarsonemus latus (Banks) en culture cotonnière. Cot Fib Trop 51(1):31–43

[CR66] van Houten Y, Knapp M, Hoogerbrugge H, Bolckmans K (2013) The potential of Amblyseius swirskii as biocontrol agent for Aculops lycopersici on tomatoes. The potential of Amblyseius swirskii as biocontrol agent for Aculops lycopersici on tomatoes. 93:51–57

[CR67] van Maanen R, Vila E, Sabelis MW, Janssen A (2010) Biological control of broad mites (*Polyphagotarsonemus latus*) with the generalist predator *Amblyseius swirskii*. Exp Appl Acarol 52(1):29–34 20191312 10.1007/s10493-010-9343-2 PMC2914298

[CR68] Zahid M, Bashir MH, Khan BS, Shahid M (2017) Toxicity of some selected pesticides against Neoseiulus barkeri (Acari: Phytoseiidae) under laboratory conditions. Pakistan J Zool, 49 (1)

[CR69] Zannou I, de Moraes G, Ueckermann E, Oliveira A, Yaninek J, Hanna R (2006) Phytoseiid mites of the genus *Neoseiulus* Hughes (Acari: Phytoseiidae) from sub-Saharan Africa. Int J Acarol 32(3):241–276

[CR70] Zannou I, Moraes G, Ueckermann E, Oliveira A, Yaninek J, Hanna R (2007) Phytoseiid mites of the subtribe Amblyseiina (Acari: Phytoseiidae: Amblyseiini) from sub-Saharan Africa. Zootaxa. 10.11646/zootaxa.1550.1.1

[CR71] Zhang Y, Xu D, Zhang Y, Wu Q, Xie W, Guo Z, Wang S (2022) Frequencies and mechanisms of pesticide resistance in *Tetranychus urticae* field populations in China. Insect Sci 29(3):827–839 34309214 10.1111/1744-7917.12957

