# Supplementary material for: Tetranychus evansi (Tetranychidae) spider mites now a major solanaceous crop pest in Côte d’Ivoire
Source: Exp Appl Acarol. 2026 Mar 14;96(3):35. doi: 10.1007/s10493-026-01125-y (PMC12988908; doi:10.1007/s10493-026-01125-y)
Supplement: Supplementary file 1 — Supplementary Material 1 [file 10493_2026_1125_MOESM1_ESM.pdf]

Survey Questionnaire – Market Gardeners, Côte d'Ivoire

Survey form No.: _________

1. Survey identification

Preliminary survey information

1.1. Survey area: _________________

1.2. Survey location: _________________ 1.3. Survey date: _________________

1.4. GPS coordinates: Long _______ Lat _______

1.5. Start time: _________ 1.6. End time: _________

1.7. Interview language: ◻ French ◻ Other (specify): _________

1.8. Presence of a translator: ◻ Yes ◻ No

1.9. Respondent contact: _________________

1.10. Enumerator name and contact: _________________

Survey conditions

1.11. Noisy environment: ◻ Yes ◻ No

1.12. Respondent agitated / poorly focused: ◻ Yes ◻ No

2. Study plot

Plot code: _________

2.1. Description of surrounding natural vegetation:

____

2.2. Main vegetable crop:

◻ Tomato ◻ Eggplant ◻ Pepper ◻ Cucumber ◻ Zucchini ◻ Beans ◻ Okra ◻ Lettuce ◻ Onion ◻ Amaranth

Variety	Development stage	Associated crop(s) (species or variety)	Transplanting date

2.3. Weed control method: ◻ Mechanical ◻ Chemical ◻ Mulching ◻ Cover crops ◻ Burning

2.4. Type of mulch and/or cover crop: _________________

2.5. Last weeding / tillage: _________ days ago

2.6. Type of weeding / tillage: ________________

2.7. Average weed cover measured: ________ %

2.8. Average weed height measured: ________ cm

2.9. Do you use plant protection products? ◻ Yes ◻ No

2.10. If yes, which product(s) were used during the last treatment?

Product No.	Frequency between treatments	Product name and concentration	Active ingredient name and dose (g/L)	Date of last treatment

2.11. Sprinkler irrigation: ◻ Yes ◻ No

2.12. Last irrigation or last rainfall: ________ days ago

2.13. Do you have anything to add?

____

2.14. Schematic representation of the plot:
