# Supplementary figures and images for: Tetranychus evansi (Tetranychidae) spider mites now a major solanaceous crop pest in Côte d’Ivoire
Source: Exp Appl Acarol. 2026 Mar 14;96(3):35. doi: 10.1007/s10493-026-01125-y (PMC12988908; doi:10.1007/s10493-026-01125-y)

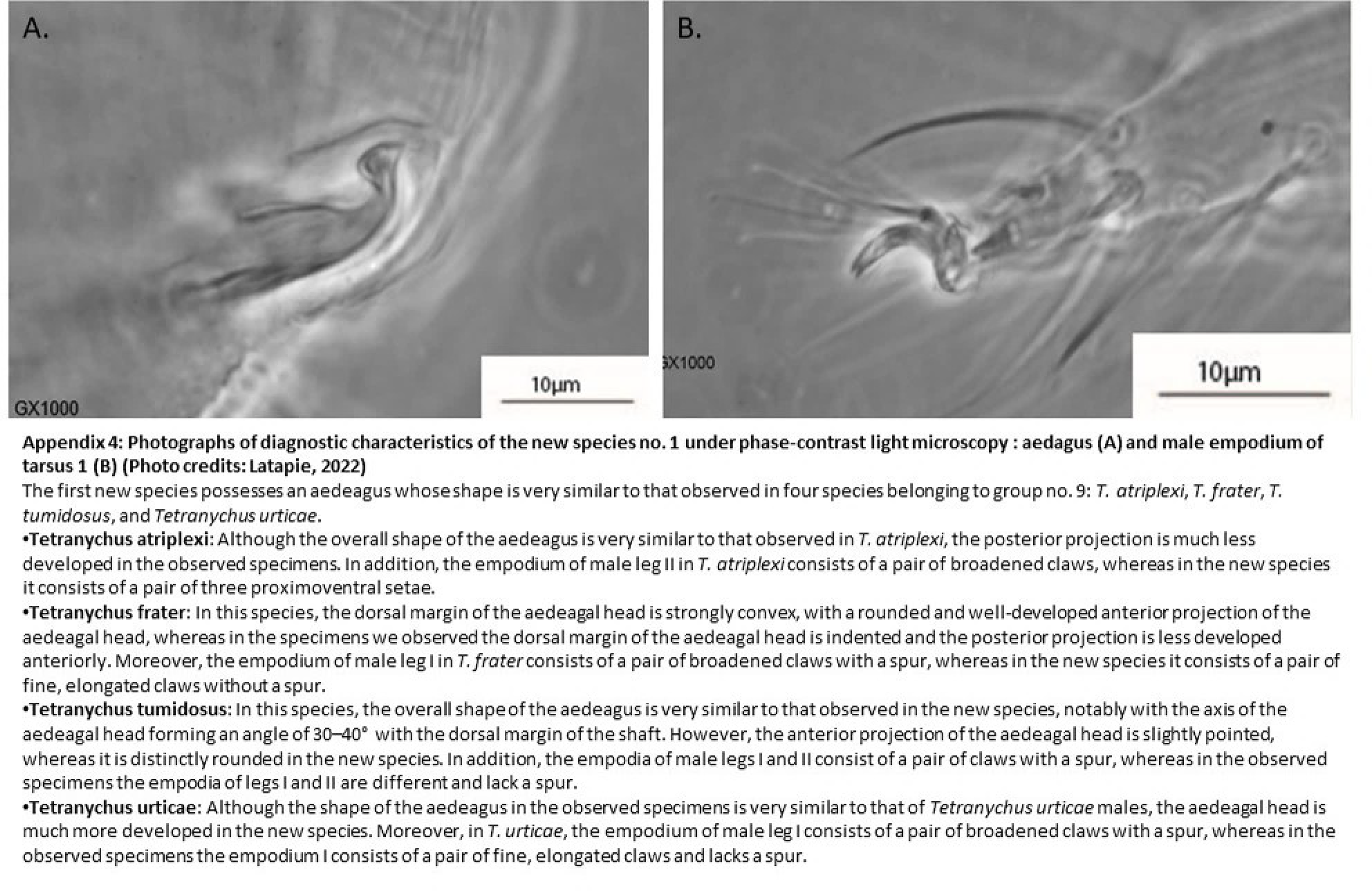

Supplement: Supplementary file 2 — Supplementary Material 2 [file 10493_2026_1125_MOESM2_ESM.tif]

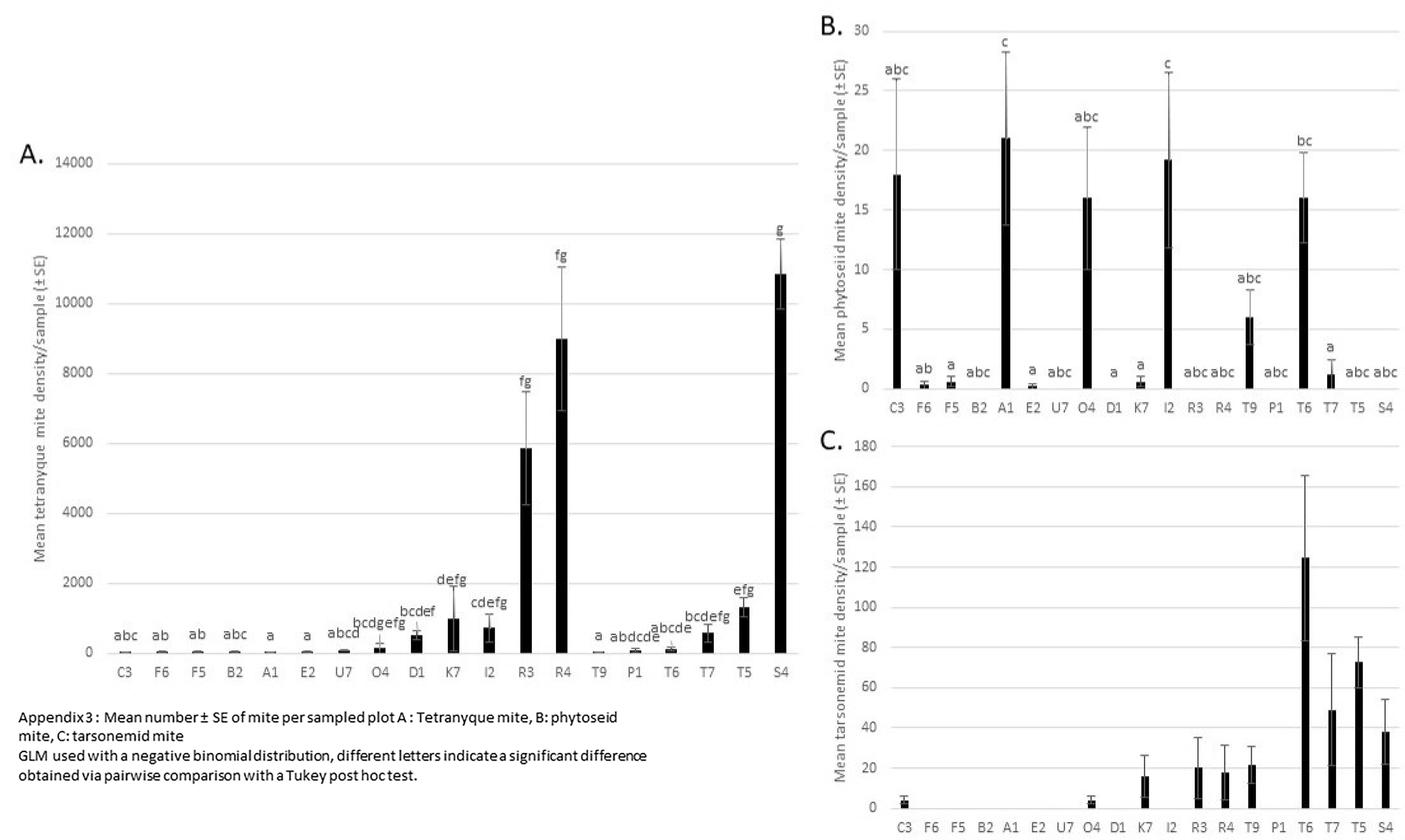

Supplement: Supplementary file 3 — Supplementary Material 3 [file 10493_2026_1125_MOESM3_ESM.tif]

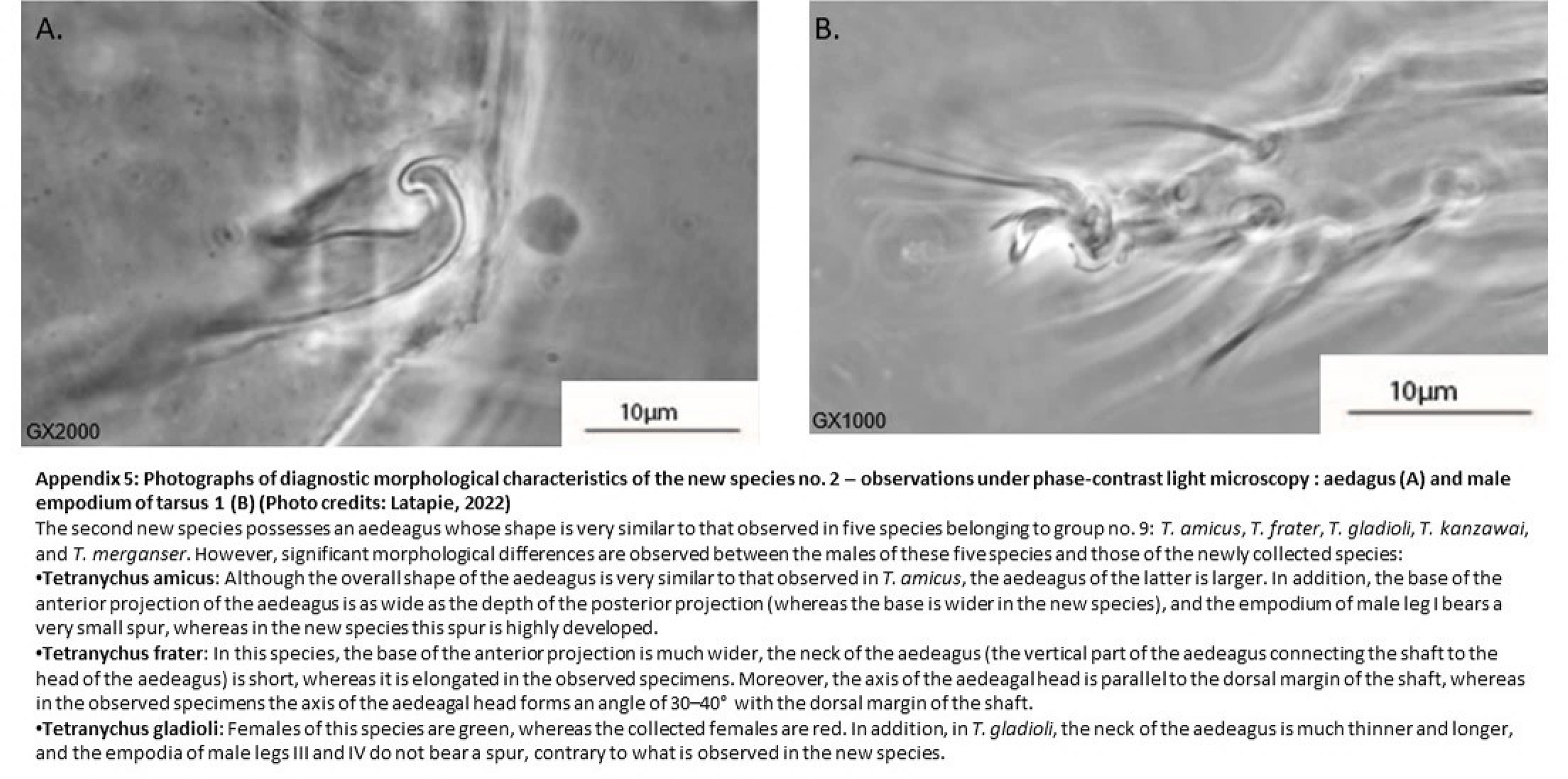

Supplement: Supplementary file 5 — Supplementary Material 5 [file 10493_2026_1125_MOESM5_ESM.tif]
