# Supplementary material for: Tetranychus evansi (Tetranychidae) spider mites now a major solanaceous crop pest in Côte d’Ivoire
Source: Exp Appl Acarol. 2026 Mar 14;96(3):35. doi: 10.1007/s10493-026-01125-y (PMC12988908; doi:10.1007/s10493-026-01125-y)
Supplement: Supplementary file 4 — Supplementary Material 4 [file 10493_2026_1125_MOESM4_ESM.pdf]

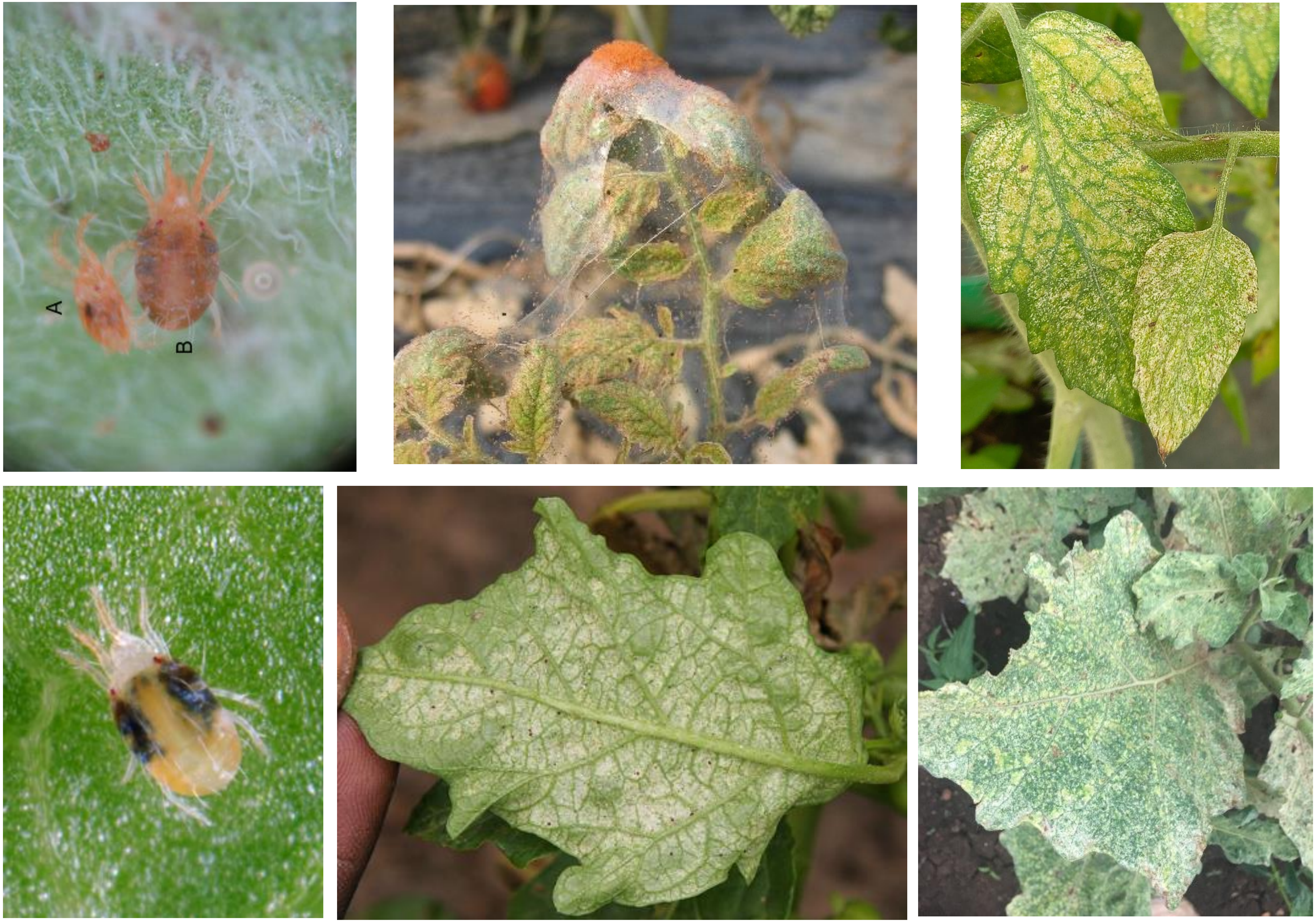

Appendix 2 : Pictures of *Tetranychus evansi*, *Tetranychus urticae*, and spider mites symptoms on tomato and eggplants used in the farmers interviews
